# Case Series of Severe Neurologic Sequelae of Ebola Virus Disease during Epidemic, Sierra Leone

**DOI:** 10.3201/eid2408.171367

**Published:** 2018-08

**Authors:** Patrick J. Howlett, Anna R. Walder, Durodami R. Lisk, Felicity Fitzgerald, Stephen Sevalie, Marta Lado, Abdul N’jai, Colin S. Brown, Foday Sahr, Foday Sesay, Jonathon M. Read, Paul J. Steptoe, Nicholas A.V. Beare, Reena Dwivedi, Marylou Solbrig, Gibrilla F. Deen, Tom Solomon, Malcolm G. Semple, Janet T. Scott

**Affiliations:** King's College London & King's Health Partners, London, UK (P.J. Howlett, A.R. Walder, M. Lado, C.S. Brown);; University of Sierra Leone, Freetown, Sierra Leone (D.R. Lisk, A. N’jai, G.F. Deen);; University College London Great Ormond Street Institute of Child Health, London (F. Fitzgerald);; Save the Children, United Kingdom and Sierra Leone, London (F. Fitzgerald);; University of Nairobi, Nairobi, Kenya (S. Sevalie);; 34th Military Hospital, Republic of Sierra Leone Joint Armed Forces Joint Medical Unit, Freetown (S. Sevalie, F. Sahr, F. Sesay);; Lancaster University, Lancaster, UK (J.M. Read);; University of Liverpool, Liverpool, UK (J.M. Read, P.J. Steptoe, N.A.V. Beare, M.G. Semple, J.T. Scott);; Royal Liverpool University Hospital, Liverpool (N.A.V. Beare, R. Dwivedi);; University of Manitoba, Winnipeg, Manitoba, Canada (M. Solbrig);; Institute of Global Health, Walton Centre NHS Foundation Trust, Liverpool (T. Solomon)

**Keywords:** Ebola, viruses, viral sequelae, neurologic, meningoencephalitis, choriomeningioencephalitis, meningitis, psychiatric, ophthalmic, migraine, 34 Military Hospital, 34th Military Hospital, Military Hospital 34, 34MH, Freetown, Sierra Leone, West Africa

## Abstract

We describe a case series of 35 Ebola virus disease (EVD) survivors during the epidemic in West Africa who had neurologic and accompanying psychiatric sequelae. Survivors meeting neurologic criteria were invited from a cohort of 361 EVD survivors to attend a preliminary clinic. Those whose severe neurologic features were documented in the preliminary clinic were referred for specialist neurologic evaluation, ophthalmologic examination, and psychiatric assessment. Of 35 survivors with neurologic sequelae, 13 had migraine headache, 2 stroke, 2 peripheral sensory neuropathy, and 2 peripheral nerve lesions. Of brain computed tomography scans of 17 patients, 3 showed cerebral and/or cerebellar atrophy and 2 confirmed strokes. Sixteen patients required mental health followup; psychiatric disorders were diagnosed in 5. The 10 patients who experienced greatest disability had co-existing physical and mental health conditions. EVD survivors may have ongoing central and peripheral nervous system disorders, including previously unrecognized migraine headaches and stroke.

The 2014–2016 West Africa Ebola virus disease (EVD) epidemic resulted in an estimated 3,956 deaths and 10,168 survivors in Sierra Leone (*1*). The use of high-quality specialty services by Ebola survivors offers an opportunity to improve understanding of debilitating post-EVD sequelae.

Central nervous system (CNS) viral invasion by EVD had been suspected but unproven until the West Africa EVD epidemic. In this outbreak, individual case-patient reports describe clinical features of meningoencephalitis or meningitis during and after acute Ebola virus (EBOV) infection, accompanied by EBOV PCR results in nonbloodstained cerebrospinal fluid samples (CSF) (*2*–*6*). Cranial imaging of 3 encephalitic patients documented changes consistent with cerebral atrophy (*3*), meningoencephalitis (*4*), and areas of diffusion restriction suggesting ischemia (*4*,*5*). Nonhuman primate EVD models and human Marburg neuropathology found EBOV-immunoreactive glial nodules and perivascular infiltrates (*7*–*9*) and evidence of choriomeningioencephalitis (*10*). In addition, a novel retinal lesion in Ebola survivors that appears to follow ganglion cell axons as they exit the optic nerve has been described (*11*). Combined with the observation that human CSF can be EBOV PCR–positive after plasma testing shows negative results (*3*,*4*), these observations raise the possibility that infected CNS cells may have a role in persistent or recurrent neurologic disease.

Observational studies of survivors report a broad range of neuropsychiatric symptoms (*12*–*14*), including increased fatigue, diminished work capacity, and sleep disturbance (*15*,*16*). Psychosocial distress caused by bereavement, stress, and stigma and formal psychiatric diagnoses of depression, anxiety, and adjustment disorder have been reported (*17*–*21*).

To define the full spectrum of characteristics and severity of neurologic and psychiatric disease, we investigated neurologic sequelae in patients with neurologic symptoms by providing specialist neurologic evaluation, psychiatric and disability assessment, and brain computed tomography (CT) imaging and retinal imaging to an EVD survivor cohort. Our additional objective was to describe psychiatric, disability, and ophthalmic outcomes for survivors with neurologic sequelae.

## Materials and Methods

We completed this prospective observational study during February 4–May 10, 2016. Patients eligible for inclusion were >12 years of age, had complete clinical records, and attended the 34 Military Hospital (34MH) Ebola Survivors Clinic, Freetown, Sierra Leone. All patients provided Ebola survivor discharge certificates as proof of identity at initial enrolment in the 34MH cohort and on attending the preliminary clinic. Furthermore, staff at the 34MH clinic had provided care in the 34MH emergency treatment unit (ETU) and could certify the validity of survivors. The preliminary clinic took place at the 34MH Ebola Survivors clinic and the specialist clinics at Connaught Hospital, Freetown, Sierra Leone.

Patients were invited to the preliminary clinic on the basis of having reported >1 major or >2 minor criteria (Table 1). These criteria were selected to maximize sensitivity for neurologic and psychiatric conditions. In addition, clinic staff invited additional patients suspected of having neurologic symptoms.

**Table 1:** Symptom-based criteria used to select patients for assessment in study of severe neurologic sequelae among Ebola virus disease survivors, Sierre Leone

Major selection criteria	Minor selection criteria
Focal weakness	Headache
Tremor	Insomnia
Altered sensation	Weakness
Vision loss	Loss of appetite
Deafness	Blurred vision
Anxiety	Dizziness
Confusion	
Depression	
Psychosis	
Inability to balance	
Auditory disturbance	
Tinnitus	
Double vision	


*Patients were selected for inclusion in a preliminary clinic examination if they exhibited >1 major or >2 minor criteria.

In the preliminary clinic, an intern physician, supported by trained nursing staff, obtained informed written consent to publish clinical data and images and administered an initial questionnaire. Further history and examination, including full neurologic examination, were accomplished by 2 physicians who used structured data recording forms. Patients with prominent or disabling symptoms of neurologic origin that required referral to the joint neurologic and psychiatric clinic were defined as having severe neurologic features. Patients with neurologic sequelae who did not warrant referral became a no severe neurologic features group. Laboratory tests, including lumbar puncture and brain CT, were available according to clinical need. Patients who had >2 psychiatric symptoms were referred for psychiatric assessment.

In the specialist clinic, full neurologic history and examination were performed individually or jointly by 2 consultant neurologists. Psychiatric assessment was performed onsite by 2 higher-level psychiatry trainees. Psychiatric assessment included Mini International Neuropsychiatric Interview (MINI-plus) and Mini Mental State Examination (MMSE; Mapi Research Trust PROVIDE, Lyon, France) and the World Health Organization Disability Assessment Schedule 2.0 (WHO-DAS 2.0; http://www.who.int/classifications/icf/whodasii/en/). The WHO-DAS 2.0 is a cross-cultural and validated tool providing a score that is compared to population percentile values (*22*). Although no cognitive or psychiatric assessment tools have been validated for the Sierra Leone population, the MMSE is frequently used by staff in the Connaught mental health clinic. Patient follow-up occurred at a second neurology clinic, in their local mental health clinic, and by telephone.

Patients underwent enhanced axial CT imaging of the brain, and scans were reviewed by a consultant neuroradiologist by using Mango software (http://ric.uthscsa.edu/mango/). All patients reviewed by specialists were invited for ophthalmologic examination, including retinal imaging. Images were reported by ophthalmologists.

## Statistical Analysis

We collected data on paper forms structured for clinical use, entered it into Microsoft Excel 2011 (Microsoft, Redmond, WA, USA), and edited it for missing information. We analyzed data by using Stata version 14.0 (StataCorp LLC, College Station, TX, USA). For sample sizes ≥35, we calculated 95% CIs for proportions by using an exact binomial method. Unadjusted odds ratios were calculated for binary and ordinal variables. We used the Wilcoxon rank sum test for comparison of continuous data and the Fisher exact test for categorical data. For multivariable logistic regression of factors associated with attending or not attending the preliminary clinic, we used a predetermined model with age (linear term), sex, and presence of major or minor criteria as explanatory variables. EBOV PCR cycle threshold (C_t_) (a figure inversely representative of plasma viral load, with >40 cycles used as a negative cutoff value) was not included in the regression models because different laboratories used different thresholds.

This study was reviewed in accordance with University of Liverpool human subjects review procedures and determined to be a nonresearch public health response activity. Ethics approval was confirmed in writing from the Sierra Leone Ethics and Scientific Review Committee. All data collection instruments were stored in a secured location, accessible only by study staff. Personal identifiers were removed from the database before analysis.

## Results

**Figure 1:**
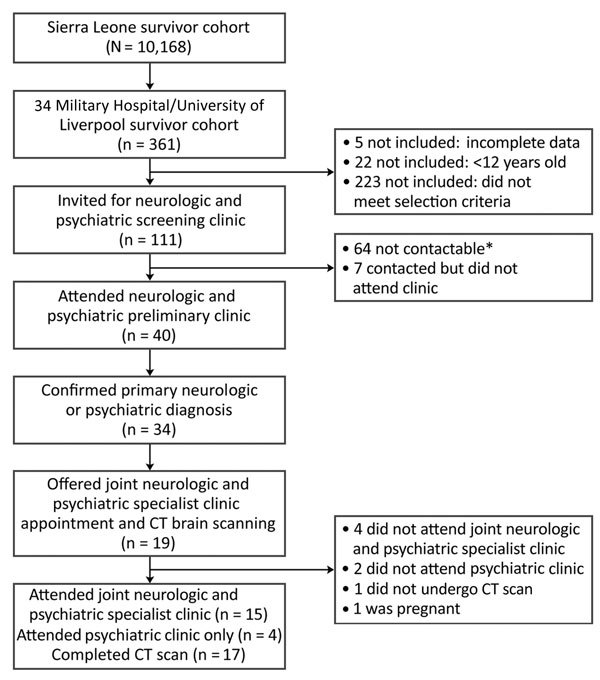
Flowchart showing clinic referral process from initial patient cohort to preliminary clinic and then specialist clinics in study of severe neurologic sequelae among Ebola virus disease survivors, Sierre Leone. Criteria for selection for preliminary clinic assessment from the 34 Military Hospital/University of Liverpool cohort were presence of >1 major or >2 minor criteria (see Table 1) or nurse-led selection on the basis of symptoms. CT, computed tomography. *Indicates telephone number was not available or telephone was repeatedly switched off.

**Figure 2:**
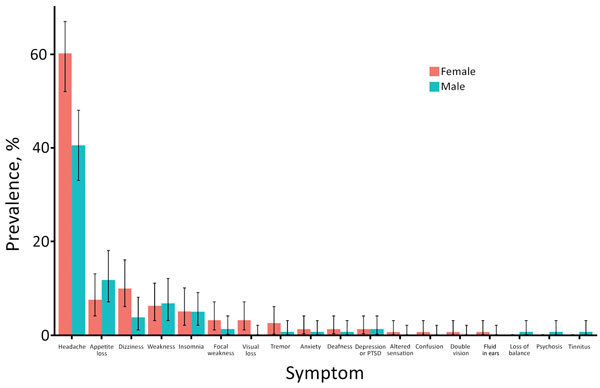
Prevalence of neurological symptoms by sex in study of severe neurologic sequelae among Ebola virus disease survivors, Sierre Leone. Cohort consisted of 24 survivors attending the 34 Military Hospital /University of Liverpool survivors clinic. Error bars indicate 95% CI. PTSD, posttraumatic stress disorders.

Of 361 patients, 5 patients were excluded because clinical data were incomplete and 22 because they were <12 years of age. Of the 334 included patients, 161 (49.7%, 95% CI 44.1%–55.3%) were female and 163 (50.3%, 95% CI 44.7%–55.9%) male; sex was not recorded for 10 patients. Median patient age was 28 (IQR 23.0–37.0) years. A total of 111 (33.2%, 95% CI 28.2%–38.6%) patients were eligible for the preliminary clinic; 32 (9.6%, 95% CI 6.6%–13.3%) patients had 1 major criteria, 74 (22.2%, CI 95% 17.8%–27.0%) had >2 minor criteria, and 12 (3.3%, 95% CI 1.7%–5.8%) were referred by clinic staff. A total of 40 (12.0%, 95% CI 8.7%–15.9%) patients attended the clinic (Figure 1). Among the 334 patients evaluated, the most common symptoms were headache (167, 50.0%, 95% CI 44.5%–55.5%), loss of appetite (33, 9.9%, 95% CI 6.9%–13.6%), and generalized weakness (22, 6.6%, 95% CI 4.2%–9.8%) (Figure 2). Female patients were more likely to be invited to the preliminary clinic than were male patients (OR 2.01, 95% CI 1.22–3.32; p = 0.03) (Technical Appendix Table 1). In those invited to the preliminary clinic, on multivariable analysis, the presence of minor criteria was associated with nonattendance (OR 0.10, 95% CI 0.03–0.56; p = 0.005) (Technical Appendix Table 2).

Of the 40 patients attending the preliminary clinic, 26 (65%, 95% CI 48.3%–79.3%) were female, and the median age was 32 (IQR 25–43) years. Patients were seen in the clinic a median of 430 (IQR 401–473) days after the first positive diagnostic results. At the time of preliminary clinic, 35 (87.5%, 95% CI 73.2%–95.8%) had neurologic or psychiatric symptoms (Table 2). None reported any substantial medical history of neurologic or mental health disorder. Of the 40 patients, 19 (47.5%, 95% CI 31.5%–63.9%) were defined as having severe neurologic signs and symptoms and were offered referral to the joint neurology and psychiatric clinic, brain CT, and retinal imaging. An additional 5 patients were referred for psychiatric review only. We found no significant difference in demographic or acute EVD features between patients with and without severe neurologic features (Table 3). A greater proportion of patients with severe neurologic symptoms were unconscious during any point in admission to the ETU, but this association was weak (OR 3.32, 95% CI 0.79–15.40; p = 0.11). Due to data sparsity, multivariable analysis was not performed.

**Table 2:** Demographics, diagnoses, and management and outcome of 35 Ebola virus disease case-patients in whom neurologic and psychiatric conditions were diagnosed at preliminary and specialist neurology and psychiatric clinics, Sierre Leone*

Patient no.	Age, y/sex	Diagnoses	Management and outcome
1	21/M	Migraine headache, psychosocial issues	MH follow-up
2	47/M	Resolved migraine headache, left retinal detachment	Review at 1 y: no change in symptoms
4	33/M	Migraine headache	DNA specialist clinic
5	54/F	Psychosocial issues, undifferentiated headache	Referred to psychiatry for assessment but did not attend
6	18/F	Undifferentiated headache	Referred return to general survivor’s clinic
7	21/F	Tension-type headache, major depressive disorder	Local MH follow-up
8	29/F	Undifferentiated headache	Referred return to general survivor’s clinic
9	26/F	Migraine headache	Referred to MH for assessment but did not attend Review at 1 y: improvement in symptoms
10	27/F	Right brachial plexus neuropathy	Physiotherapy. Review at 1 y: substantial improvement in weakness
11	42/F	Right striatocapsular infarct, generalized anxiety disorder	Physiotherapy, MH follow-up
13	58/F	Undifferentiated headache	Referred return to general survivor’s clinic for nonneurologic and other symptoms
14	38/M	Possible anterior uveitis, undifferentiated headache	Ophthalmology referral
15	49/F	Tension-type headache	Referred return to general survivor’s clinic for nonneurologic symptoms
16	31/F	Migraine headache	Propranolol 20 mg/d; symptoms improved (unable to quantify)
17	51/F	Undifferentiated headache, peripheral sensory neuropathy	Referred return to general survivor’s clinic for nonneurologic symptoms
18	32/F	Tinnitus, anterior uveitis	Ophthalmology referral. MH follow-up. Review at 1 y: improvement in tinnitus, now occasional
19	38/M	Undifferentiated headache	Local MH follow-up
20	30/F	Resolved migraine headache	Review at 1 y: new onset headache with cluster-type features
21	32/F	Migraine headache, right eye cataract, tinnitus	Ophthalmology referral
22	21/F	Migraine headache, tinnitus	Propranolol 20 mg/d. Headache improved from 8/10 to 4/10. Review at 1 y: no further headache
23	46/M	Essential tremor, undifferentiated headache	DNA specialist clinic
24	43/F	Migraine headache	Propranolol 20 mg/d, initially 10/10 headache pain now better (not able to quantify). Review at 1 y: decreased frequency of headaches, now occasional
25	42/M	Extensive right MCA infarct, major depressive disorder	Physiotherapy, MH follow-up. Review at 1 y: improvement in symptoms. Patient subsequently died.
26	25/F	Ulnar nerve palsy	DNA specialist clinic
27	25/M	Migraine headache, asymmetric lower limb muscle wasting	MH follow-up. Review at 1 y: decreased frequency of headaches, now occasional
28	21/F	Tension-type headache	Review at 1 y: decreased frequency of headaches; now occasional. Fever/rash during pregnancy; miscarriage
29	61/F	Migraine headache, bilateral cataract	Local MH follow-up
30	19/F	Anterior uveitis, undifferentiated headache	Urgent referral to local ophthalmology clinic
31	33/F	Migraine headache, generalized anxiety disorder	Propranolol 20 m/d; improved headache from 10/10 to 6/10. MH follow-up
32	43/F	Undifferentiated headache, arthralgia	Referred to local ophthalmology clinic
33	41/F	Migraine headache, anxiety	MH follow-up, simple analgesia. Review at 1 y: decreased frequency of headaches, now occasional
34	25/F	Undifferentiated headache	Referred to general survivor’s clinic
35	35/M	Migraine headache, asymmetric sensory peripheral neuropathy, major depressive disorder	MH follow-up, propranolol 20 mg/d, gabapentin 300 mg each night; diet advice and review in diabetic clinic referral. Headache improved (unable to quantify); pain in feet improved. Review at 1 y: decreased frequency of headaches, now occasional; improvement in neuropathy
37	12/F	Severe neurocognitive impairment, postviral encephalitis	Referral to orphanage for 24-h care
38	21/M	Undifferentiated headache, arthralgia	ND
*MH, mental health; MCA, middle cerebral artery; ND, no data.			

**Table 3:** Demographics, clinical characteristics during acute admission, and cycle threshold of preliminary clinic group in study of severe neurologic sequelae among Ebola virus disease survivors, by those who had severe and those who had no severe neurologic conditions, Sierre Leone*

Characteristic	No severe neurologic features, n = 21	Severe neurologic features, n = 19	Crude odds ratio† (95% CI)
Age, y, median (IQR)	28 (23–60)	32 (25–42)	0.01 (0.00–0.036)/y
Female sex, % (95% CI)	48 (43–54)	68 43–87	2.3 (0.79–7.60)
Length of stay, d, median (IQR)	18 (14–28)	25 (13–29)	0.02/d
Seizures during admission, % (95% CI)	19 (5–42)	21 (6–46)	1.13 (0.18–7.23)
Unconscious during admission, % (95% CI)	33 (15–57)	63 (38–83)	3.32 (0.79–15.4)
Bleeding during admission, % (95% CI)	19 (5–42)	5 (0.1–26)	0.24 (0.00–2.80)
Cycle threshold, median (IQR)	22.8 (22.1–24.1), n = 9	27.2 (22.5–30.1), n = 10	0.22 (0.7–1.3) for each increment

*Severe conditions were those requiring specialist referral. †Odds ratio of patients having severe neurologic features compared with those who did not.

## Clinical Features

In the preliminary clinic, a new or different headache since acute EVD admission was reported by 30 (75.0%, 95% CI 58.8%–87.3%) patients; female:male ratio was 2:1. Of those with headache, 14 (46.6%, 95% CI 38.3%–65.7%) had undifferentiated headache, 13 (43.3%, 95% CI 25.5%–62.6%) migraine, and 3 (10.0%, 95% CI 2.1%–26.5%) tension-type headaches (Technical Appendix Table 3). Five patients who had migraine headaches were prescribed oral propranolol (20 mg 1×/d), in keeping with WHO guidance on survivor care (*23*); 4 returned for follow-up 1 month after treatment and reported symptomatic improvement.

One male and 1 female survivor, both 42 years of age, had evidence of stroke; symptom onset occurred at the time of acute EVD. These patients had the highest disability scores (WHO Disability Assessment Schedule 2.0 scores 89.58 and 33.33, respectively) and met criteria for a mental health disorder (see Case Study 1). Given the major vessel territory distribution on CT, these strokes are suspected to be mature ischemic infarcts.

Two survivors had peripheral sensory neuropathy and 2 focal peripheral nerve lesions. Brachial plexopathy was diagnosed in a 27-year-old woman during acute EVD. Neuropathy screening of the patient for treatable causes was negative, and she was referred for physiotherapy. Asymmetric glove and stocking peripheral sensory neuropathy was diagnosed in a 35-year-old man, occurring since ETU discharge. Diabetes and major depressive disorder were diagnosed, and he was referred to the diabetes and mental health clinic. Other reported neurologic symptoms in the cohort included 3 cases of tinnitus, 2 cases of tremor, and 1 case of asymmetric lower limb atrophy with weakness of unknown etiology. Of the 19 patients who attended the specialist clinic, 12 were reviewed 1 year later, in June 2017; 10 reported improvement of symptoms, 1 reported no changes, and 1 reported a new headache. After this, case-study patient 1 died.

Psychiatric symptoms were common among 21 (52.5%, 95% CI 36.1%–68.4%) survivors describing difficulty sleeping; 12 (30.0%, 95% CI 16.5%–46.5%) described depressive symptoms and 11/40 (27.5%, 95% CI 14.6%–43.9%) anxiety symptoms (Technical Appendix Table 4). Of 24 (60.0%, 95% CI 43.3%–75.1%) survivors referred for psychiatric review, 19 (47.5%, 95% CI 31.5–63.8%) attended the clinic. Of those, 16 (63.3%) required referral for local mental health follow-up, of whom 5 met criteria for mental disorder (2 generalized anxiety disorder and 3 major depressive disorder). The most common reasons for mental health referral were stigma, grief, and loss of employment. Of the 19 patients who attended the psychiatric clinic, median MMSE score was 93.3% (IQR 87.7%–96.3%). No patient reported suicidal ideation.

Among 19 survivors assessed for disability, the median WHO-DAS 2.0 score was 8.3% (IQR 3.1%–13.5%) corresponding to the 69th percentile of the normative population. The 9 patients who had a disability score >10 (corresponding to scores found in <27.65% of the normative population) included all survivors affected by mental health disorders, stroke, and peripheral neuropathies for which disabilities were assessed. The most severe case of disability is described in Case Study 2.

**Figure 3:**
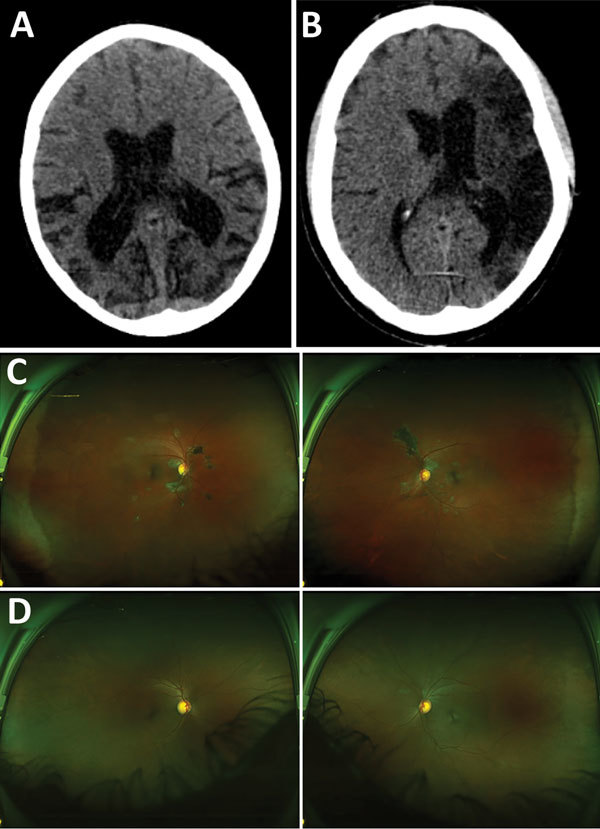
Representative nonenhanced computed tomography (CT) brain scans and composite scanning laser ophthalmoscope fundus images of 2 Ebola virus disease survivors attending a joint neurologic and psychiatric clinic in Sierre Leone. A) Patient no. 37, female, age 12. CT of brain shows disproportionate parietal and temporal lobe atrophy. B) Patient no. 25, male, age 42. CT of brain shows extensive gliosis within the left middle cerebral artery territory reflects an old infarct with ex-vacuo dilatation of left lateral ventricle due to hemispheric volume loss. C) Patient no. 12, age 40. Retinal imaging shows left and right eye, with extensive bilateral peripapillary pale retinal lesions with pigmentation of larger lesions. Lesions appear to spare the fovea. Visual acuity was 20/25 (right) and 20/20 (left) (*24*). D) Patient no. 25, male, age 42. Retinal imaging shows left and right eye, with peripapillary pale retinal lesions. Visual acuity was 20/25 in both eyes (*25*).

Of 17 patients who underwent brain CT, abnormalities were shown for 7. Three scans showed evidence of cerebral or cerebellar atrophy that was atypical for patient age (Figure 3, panel A), 2 confirmed the clinical assessment of stroke (Figure 3, panel B), and 2 showed evidence of calcification, differentials of which include previous focal hemorrhage occurring >1 year before the scan.

Of the 40 survivors evaluated at the prelimnary clinic, 12 described eye pain (30.0%, 95% CI 16.6%–46.5%) and 8 (20.0%, 95% CI 9.1%–35.6%) described partial visual loss. Of 17 patients who attended the ophthalmology specialist clinic for examination, and wide field-scanning laser ophthalmoscope imaging, 3 (15.8%) had Ebola retinal lesions (Figure 3, panels C) (*11*). One survivor had unilateral retinal detachment, 1 intermediate uveitis, and 1 posterior subcapsular cataract suggestive of previous uveitis.

## Case Studies

### Case Study 1—Patient No. 25

Patient no. 25 was a previously fit and well 41-year-old male soldier who had an uncomplicated 8-day acute admission to a hospital for treatment of EVD; 3 days after discharge, he had sudden onset of left-sided weakness and dysphasia. In the neurology clinic, 545 days after his admission for acute illness, examination was consistent with a right upper motor neuron lesion. His MMSE was 26/27 and WHO-DAS 2.0 score 89.58, conforming to significant disability. He exhibited a pervasive low mood, anhedonia, feelings of worthlessness, guilt, frustration, and hopelessness regarding the future because of disability. His CT results showed extensive gliosis within the left middle cerebral artery territory, in keeping with mature infarct (Figure 3, panel B.). Retinal imaging showed bilateral Ebola retinal lesions (Figure 3, panels C, D [*24*]). Stroke and major depressive disorder were diagnosed. He was referred for physiotherapy, which resulted in marked improvement in symptoms, and received mental health clinic follow-up. Approximately 1 year after the intial clinic visit, the patient had an undifferentiated fever, serum from a blood sample tested EBOV PCR negative, but he died several days later.

### Case Study 2—Patient No. 37

A 12-year-old girl who had a normal developmental history had a C_t_ of 27.9 at hospital admission for EVD; she improved with treatment and became serum EBOV PCR negative on days 15 and 17. On day 20, her consciousness level gradually declined and fever recurred; she then had recurrent seizures for 48 hours that were partially controlled by administration of phenytoin and diazepam. Her consciousness level gradually improved over the next 4 weeks to spontaneously alert but confused. At the preliminary clinic, 454 days after acute admission, she was blind and had substantial hearing loss and severe cognitive impairment. She was doubly incontinent and required 24-hour care for all activities of daily living. Her CT results showed disproportionate parietal and temporal lobe atrophy (Figure 3, panel C). CSF test results were EBOV negative; results of a specialist’s ophthalmology review were unremarkable. Planning for her complex care needs required multiagency and multidisciplinary coordination to find an orphanage and provide resources and training to that facility to help manage her needs. She was unable to attend the specialist neurology clinic because of the remote location of her orphanage. Follow-up visits to the orphanage from the medical, psychiatric, and therapies team found no major functional improvements.

## Discussion

Previous studies have outlined the frequency of a variety of neurologic symptoms in EVD survivors (*13*). Our specialist case series from the 34MH survivor’s cohort confirms the presence of central and peripheral nervous system disorders and found these to be associated with a broad range of disability. The most frequent neurologic diagnosis was migraine headaches; the next most common, respectively, were stroke, peripheral sensory neuropathy, and focal peripheral nerve lesions. Most survivors had co-occurring mental health problems, the most frequent psychiatric diagnoses being major depressive disorder and generalized anxiety disorder. The most severely affected patients had symptoms of blindness, deafness, focal weakness, and cognitive dysfunction associated with disability and mental illness.

The diagnosis of migraine headache found in 13 case-patients was characterized by intermittent, throbbing headaches associated with photophobia, phonophobia, and, in some cases, vomiting. These symptoms were either new or substantially worse after acute EVD. In a small group, treatment with propranolol according to WHO guidelines (*23*) led to subjective improvement. To date, headaches in the EVD survivor population have not been well described; a small group of survivors was noted to have unilateral and throbbing headaches (*19*), although frequency from the 2014–2016 West Africa Ebola disease outbreak ranges 22%–68% (*14*,*19*,*20**,**25**)*, In the only case–control study in which 90% of survivors reported headache, a high prevalence of 75% in the control population meant this finding was not significant (*16*). A recent meta-analysis reported a community migraine prevalence of 5.6% (95% CI 4.6%–6.7%) in community-based studies in Africa (*26*). Because our preliminary clinic selection criteria required patients with headache to have >1 associated symptom, our headache findings and prevalence may not be representative of the survivor population. Potential mechanisms for migraine headache in EVD survivors may include autonomic dysregulation (*27*), changes in tryptophan-serotonin levels after infection (*28*), or ongoing neuroinflammation, as seen in HIV infection (*29*). With limited diagnostic methods, we are unable to determine specific etiologies of all neuropathy or suspected myopathy cases; however, diabetic neuropathy, entrapment neuropathy, or critical illness polyneuropathy with slow recovery are potential causes.

Radiologic imaging showed sequelae of focal or generalized atrophy or stroke in some patients. As previously reported (*5*,*12*), we found substantial cerebral atrophy in 2 patients and isolated cerebellar atrophy in 1 other survivor. One patient had a reported case of late onset encephalitis (*3*), and 1 patient’s imaging correlated with substantial cognitive deficit, cortical blindness, and hearing impairment (see Case Study 2). Although it is possible the atrophy was related to birth complications, nutritional deficiency, or childhood illness, the prominent parietal and temporal lobe atrophy of this adolescent case-patient resembles radiologic findings in subacute sclerosing panencephalitis, a chronic CNS infection caused by defective measles virus, raising the possibility of similar CNS mechanisms of EVD and measles or persistent CNS infection (*30*). Cerebral CT images of 2 stroke case-patients, whose neurologic symptom onset occurred during acute EVD, were consistent with ischemic stroke. Suspected stroke during acute EVD has been reported (*31*), and thromboelastography, a measurement of thrombotic tendency, done during and after acute EVD illness, suggests a prothrombotic period in the immediate aftermath of EVD (*32*).

In 3 (15.8%) of 19 patients in the severe neurologic features group, we observed the novel Ebola peripapillary retinal lesion, recently reported by Steptoe et al. (*11*), who described a similar prevalence (14.6%) among a wider survivor population. Although the most likely mechanism of CNS viral entry is from circulating infected cells, the presence of retinal peripapillary lesions, thought to represent virus spread along the retinal nerve fiber or ganglion cell axon layers, raises the possibility of CNS viral entry by neuronal spread.

The group of patients who had severe neurologic features generally had good results from adapted MMSE testing. For a patient who had a confirmed case of late-stage EVD encephalitis and initial neurocognitive impairment (*3*), assessment 1 year later showed good long-term recovery. This finding is encouraging and in keeping with 2 case reports of recovery from neurocognitive impairment (*33*). Despite onset being 1 year after acute disease and many patients having been initially referred to counselors, 5 of 19 patients met criteria for psychiatric disorder, all 19 had concurrent physical symptoms, and 16 required mental health follow-up. As previously reported, survivors cited stigma, grief, and loss of employment as major stressors impeding recovery (*17*,*34*).

A recent case–control study found survivors had major limitations of vision, cognition, affect, and, most markedly, mobility (*35*). In our study, we found 10 participants who reported high levels of disability and also had physical symptoms and co-occurring mental health issues. This clustering of physical and psychiatric sequelae and disability suggests a subset of patients most affected after acute EVD and with the greatest care needs. In the small number of self-selecting case-patients on whom we followed up 18 months after the first neurologic/psychiatric clinic, patients generally reported symptomatic improvement; however, improvement was not uniform. One case-patient subsequently died (patient no. 25; see Case Study 1) and another remains dependent for all activities of daily living (patient no. 37; see Case Study 2).

Our study observed no association between severe neurologic conditions and admission C_t_. To the contrary, among the 2 patients who had both prolonged periods of unconsciousness and cerebral atrophy on CT (patients no. 2 and 16), the neurologic episodes occurred late in the acute disease period, not at the time of peak viral load. Similarly, 2 case reports describe a prolonged meningoencephalitic stage of disease or meningoencephalitis occurring months after recovery (*4*,*5*). Of note, we found no cases of CNS infection recurrence. Unconsciousness during acute admission was more common among those who had severe neurologic symptoms on follow-up, although not to a significant degree, possibly caused by limited sample size (OR 3.32, CI 0.79–15.4; p = 0.11). Our preliminary group was selected on the basis of existing neurologic symptoms, which precludes a conclusion of causation and generalization to the wider EVD survivor population.

A major limitation of our case series is that we cannot firmly determine causation between our findings and the diagnosis of EVD beyond the temporal association. Furthermore, in keeping with other observational studies, a lack of reliable countrywide denominator data on conditions such as headache or stroke means we cannot assess the representativeness of our results. Validating our findings would require a large case–control study, in which our data could be used as a basis for study design. Retrospectively asking about acute symptoms incurs the possibility of recall bias; however, as acute records of the EBV outbreak clinics are sparse and linkage-challenging, this represented the most viable option. Despite our multiple attempts, the outcomes of 71/111 patients who were invited to but did not attend the preliminary clinic remain unknown. Although our analysis shows those with minor selection criteria were among those less likely to attend (p = 0.005), it is still possible we underrepresented patients who had more disabling conditions and were unable to access the service, as exemplified by the patient in Case Study 2. Further research should focus on a complete characterization of pathways of sequelae and persistent infection (*36*).

Our case series, supported by brain CT imaging, confirms there are long-term neurologic sequelae in EVD survivors and a substantial proportion of these patients have ongoing mental health problems and disability. Often, these issues cluster together, and services should therefore seek out and support patients with a high burden of illness. If we wish to expand specialist services to the remaining EVD survivors and broader population, the only credible and sustainable option is to greatly increase support for in-country specialist training of doctors.

Technical Appendix Additional information from the case series of severe neurologic sequelae of Ebola virus disease during the 2014–2016 epidemic in Sierra Leone. For the cohort of 334 Ebola virus disease survivors, analyses of major or minor inclusion criteria, age, and cohort clinic symptoms according to sex; comparison of patients invited who did or did not attend the preliminary clinic; data analysis of subgroup of patients with neurologic, psychiatric, and ophthalmologic diagnoses who attended preliminary and specialist neurology and psychiatric clinics; and symptom analysis.
