# Supplementary material for: Case Series of Severe Neurologic Sequelae of Ebola Virus Disease during Epidemic, Sierra Leone
Source: Emerg Infect Dis. 2018 Aug;24(8):1412–21. doi: 10.3201/eid2408.171367 (PMC6056101; doi:10.3201/eid2408.171367)
Supplement: Technical Appendix — Additional information from the case series of severe neurologic sequelae of Ebola virus disease during the 2014–2016 epidemic in Sierra Leone. For the cohort of 334 Ebola virus disease survivors, analyses of major or minor inclusion criteria, age, and cohort clinic symptoms according to sex; comparison of patients invited who did or did not attend the preliminary clinic; data analysis of subgroup of patients with neurologic, psychiatric, and ophthalmologic diagnoses who attended preliminary and specialist neurology and psychiatric clinics; and symptom analysis. [file 17-1367-Techapp-s1.pdf]

Article DOI: https://doi.org/10.3201/eid2408.171367

Case Series of Severe Neurologic Sequelae of Ebola Virus Disease during Epidemic, Sierra Leone

Technical Appendix

Technical Appendix Table 1. Univariate analysis of major or minor inclusion criteria, age, and cohort clinic symptoms according to sex in cohort of Ebola virus disease survivors (n = 334)*†‡§

Category	Invited to preliminary clinic	Not invited to preliminary clinic	Crude odds ratio (95% CI)
Fit neurology criteria for invitation*			
All	111	223	NA
M	42 (39)	123 (56)	1
F	66 (61)	96 (44)	2.01 (1.22 – 3.32)
Fit major criteria			
All	32	299	NA
M	8 (25)	155 (52)	1
F	23 (75)	138 (48)	3.25 (1.34 – 8.65)
Fit minor criteria			
All	74	260	NA
M	29 (39)	134 (52)	1
F	43 (58)	118 (46)	1.60 (0.96 – 3.00)
Invited by clinic staff			
All	12	322	NA
M	6 (50)	0 (0)	1
F	6 (50)	0 (0)	1.02 (0.27 – 3.90)
Age (years, IQR)†			
All	29 (22–38)	28 (23–36)	0.007 per +1 y (0.016–0.022)
M	29 (22–38)	27 (24–35)	NA
F	27 (23–35)	29 (22–37)	NA
Major criteria			
Focal Weakness			
All	7	NA	NA
M	2 (29)	NA	1
F	5 (71)	NA	2.58 (0.41–27.4)
Tremor			
All	5	NA	NA
M	1 (20)	NA	1
F	4 (80)	NA	4.11 (0.40–204.46)
Altered Sensation			
All	1	NA	NA
M	0	NA	NA
F	1 (100)	NA	NA
Visual loss			
All	5	NA	NA
M	0	NA	NA
F	5 (100)	NA	NA
Deafness			
All	3	NA	NA
M	1 (33)	NA	1
F	2 (66)	NA	2.04 (0.10–120.94)
Anxiety			
All	3	NA	NA
M	1 (33)	NA	1
F	2 (66)	NA	2.04 (0.10–120.9)
Confusion			
All	0	NA	NA

Category	Invited to preliminary clinic	Not invited to preliminary clinic	Crude odds ratio (95% CI)
Fit neurology criteria for invitation*			
M	0	NA	NA
F	1 (100)	NA	NA
Depression			
All	4	NA	NA
M	2 (50)	NA	1
F	2 (50)	NA	1.01 (0.073–14.12)
Psychosis			
All	1	NA	NA
M	1 (100)	NA	NA
F	0	NA	NA
Inability to balance			
All	1	NA	NA
M	1 (100)	NA	NA
F	0	NA	NA
Double vision			
All	1	NA	NA
M	0	NA	NA
F	1 (100)	NA	NA
Tinnitus			
All	1	NA	NA
M	1 (100)	NA	NA
F	0	NA	NA
Minor criteria‡			
	Headache		
All	74	93	NA
M	24 (32)	49 (45)	1
F	50 (68)	42 (53)	2.23 (1.39–3.56)
Insomnia			
All	13	4	NA
M	6 (46)	2 (50)	1
F	7 (54)	2 (50)	1.01 (0.32–3.18)
Weakness			
All	15	7	NA
M	6 (40)	5 (71)	1
F	9 (60)	2 (29)	0.91 (0.33–2.45)
Loss of appetite			
All	25	8	NA
M	12 (48)	7 (88)	1
F	13 (52)	1 (13)	0.61 (0.26–1.38)
Dizziness			
All	21	1	NA
M	6 (29)	0 (0)	1
F	15 (71)	1 (100)	2.88 (1.03–9.23)

*Values are no. (%) patients except as indicated.
†Sex not available for 7 patients (n = 327). F, female; M, male; NA, not applicable.
‡Age not available for 22 patients (n = 312 patients).
§Symptoms not available for additional 3 patients (n = 331).

Technical Appendix Table 2. Crude odds ratio and multivariable adjusted regression analysis comparing of patients invited and who did attend the preliminary clinic and those invited but did not attend the preliminary clinic*

Variable	Total no. (%)	Invited and attended (%)	Invited did not attend (%)	Crude OR (95% CI)	Adjusted OR (95% CI)
Total	111 (100)	40 (36)	71 (64)	NA	NA
Sex (n = 108*)					
M	42 (39)	14 (33)	28 (66)	1; 1.30 (0.53–3.19)	1.58 (0.62–4.01)
F	66 (61)	26 (39)	40 (61)		
Age, years (n = 111) Median (IQR)	29 (22–38)	33 (25–43)	27 (21–35)	0.009 (0.0018–0.018) per + 1y	0.006 (−0.10–0.27) per +1 y
Fitted major criteria†	26	13 (35)	13 (27)	1.48 (0.57–3.77)	0.34 (0.08–1.41)
Fitted minor criteria†	104	18 (49)	56 (79)	0.25 (0.10–0.65)	0.1 (0.03–0.56)

*Female/male sex not available for 3 cases; NA, not applicable.
†Data available for 108/111 patients invited to the preliminary clinic; 3 patients invited to the preliminary clinic by clinic had attended the 34MH clinic after data collection of initial review of notes had been made.

Technical Appendix Table 3. Demographics, acute presentation, cycle threshold values, neurologic features, mini-mental state examination (MMSE), WHO Disability Score 2.0 (WHODAS), CT brain findings, diagnosis and management and outcome of 35 patients with neurologic and psychiatric diagnoses, and 1 further ophthalmology diagnosis attending preliminary and specialists neurology and psychiatric clinics*

Pt no (Sex/Age)	Acute disease features (Length of stay/days) (Zaire Ebola virus PCR result/Ct value)	Neurologic features at review in clinic - time since acute disease/days, symptoms and examination findings, MMSE and WHO-DAS 2.0 score, selected serum, cerebrospinal fluid radiographic, ECG and retinal investigations	Neurologic/psychiatric diagnosis	Management and outcome
1 (M/21)	Fever, cough with sputum, sore throat, arthralgia/myalgia, intense fatigue, **headache**, **altered consciousness**, conjunctivitis, **unconsciousness** (13 d)	(438 d) Headache, throbbing frontal with associated photophobia and scotoma. Intermittent intention tremor. Difficulty sleeping and anxiety. Itchy skin over hands. MMSE 28/30. WHO-DAS 4.17	Migraine headache Fungal skin infection Psychosocial issues	Simple analgesia. Antifungal skin preparation. Local mental health follow-up.
2 (M/47)	Fever, chest pain, arthralgia/myalgia, intense fatigue, **headache**, shortness of breath, **altered consciousness**, nausea, vomiting, diarrhea, **unconscious** (11 d). C_t value – 30.3	(413 d) Intermittent all over headache, lasting up to one week, occurring approximately every 2 weeks. Resolved on review in specialist clinic. Visual disturbance – intermittent scotoma. MMSE 30/30. WHO-DAS 0. CT Brain - Relative cerebellar volume loss. Retinal imaging - Left retinal detachment. Right normal	Resolved migraine headache, arthralgia, left retinal detachment	Simple analgesia. Review at 1 y – ongoing symptoms
4 (M/33)	Fever, sore throat, chest pain, arthralgia/myalgia, intense fatigue, **headache**, **altered consciousness**, abdominal pain, conjunctivitis, rash, **unconscious** (29 d)	(409 d) Band-like headache, sometimes so severe it makes him feel confused. Lasts between 1 week to 1 d. Associated with scotoma. Retinal imaging - Normal bilaterally	Migraine headache	DNA specialist clinic
5 (F/54)	Fever, sore throat, runny nose, chest pain, joint pain, intense fatigue, **headache**, shortness of breath (26 d)	(537 d) Intermittent dizziness, altered taste in mouth, feels like burning when eats. Joint pains. All over headache with scotoma. Visual hallucinations/ flashbacks, feeling of anxiety, restlessness and irritability. On	Psychosocial issues. Symmetric large joint polyarthritis Undifferentiated headache	Referred to psychiatry for assessment but did not attend

Pt no (Sex/Age)	Acute disease features (Length of stay/days) (Zaire Ebola virus PCR result/Ct value)	Neurologic features at review in clinic - time since acute disease/days, symptoms and examination findings, MMSE and WHO-DAS 2.0 score, selected serum, cerebrospinal fluid radiographic, ECG and retinal investigations	Neurologic/psychiatric diagnosis	Management and outcome
		examination, symmetric large joint polyarthritis.		
6 (F/18)	Fever, cough, sore throat, runny nose, chest pain, arthralgia/myalgia, intense fatigue, **headache**, shortness of breath, **altered consciousness**, abdominal pain, nausea, vomiting, diarrhea, conjunctivitis, rash, skin ulcer (8 d)	(413 d) New generalized headache no associated symptoms. Joint pains and cough	Undifferentiated headache, arthralgia	Referred back to general survivor's clinic
7 (F/21)	Fever, cough, sore throat, runny nose, ear ache, chest pain, arthralgia/myalgia, **headache**, shortness of breath, **altered consciousness**, nausea, vomiting, diarrhea, conjunctivitis, rash, **unconscious** (42 d)	(480 d) Band-like headache, present most of the time (daily). Associated scotoma. Vaginal candidiasis and itchy skin. Hallucinations and feeling of anxiety. Low mood, poor sleep, anhedonia, irritability, angry outbursts, isolated, stigmatised by community. MMSE 28/29. WHO-DAS 10.42	Tension-type headache, major depressive disorder, vaginal candidiasis	Local mental health follow-up, diprobase for skin
8 (F/ 29)	Fever, hemoptysis, sore throat, earache, arthralgia/myalgia, intense fatigue, **headache**, shortness of breath, **altered consciousness**, **seizures**, abdominal pain, nausea, vomiting, diarrhea, conjunctivitis (18 d). C_t value – 19.4	(398 d) Generalized headache with no associated symptoms. Intermittent visual	Undifferentiated headache	Referred back to general survivor's clinic
9 (F/26)	Fever, heamoptysis, chest pain, joint pains, **headache**, shortness of breath, **altered consciousness**, **seizures**, abdominal pain, nausea, vomiting, diarrhea, conjunctivitis, rash (14 d). C_t value – 23.4	(394 d) Headache, frontal, nightly, little improvement with analgesia. Associated with blurred/altered vision. Bony and joint pains. Hallucinations, feelings of anxiety and restlessness	Migraine headache Arthralgia	Referred to MH for assessment but did not attend. Review at 1 y – improvement in symptoms
10 (F/27)	Fever, hemoptysis, headache, **altered consciousness**, **seizures**, nausea, vomiting, diarrhea, **unconscious** (2 d) (44 d)	(408 d) New right arm weakness since discharge. On examination, lower motor neuron weakness (3/5) and sensory impairment right upper limb. MMSE 18/21. WHO-DAS 14.58. Brain CT – Normal study. Retinal imaging - Normal bilaterally	Right brachial plexus neuropathy	Physiotherapy, analgesia. Review at 1 y - significant improvement in weakness
11 (F/42)	Fever, intense fatigue, **headache**, **altered consciousness**, nausea, vomiting, conjunctivitis, **unconscious** (33 d). C_t value – 22.1	(398 d) Right sided weakness occurred during admission now improved but still cannot write (R handed). Pervasive low mood as unable to carry out activities of daily living, anhedonia, feeling of worthlessness, tearful in clinic. On examination, right VII weakness, right upper limb; 4/5	Right striatocapsular infarct, Generalized Anxiety Disorder	Physiotherapy, MH follow up

Pt no (Sex/Age)	Acute disease features (Length of stay/days) (Zaire Ebola virus PCR result/Ct value)	Neurologic features at review in clinic - time since acute disease/days, symptoms and examination findings, MMSE and WHO-DAS 2.0 score, selected serum, cerebrospinal fluid radiographic, ECG and retinal investigations	Neurologic/psychiatric diagnosis	Management and outcome
		power, brisk reflexes, sensory impairment. Right plantar equivocal. BP 140/90. All over headache. MMSE 28/30. WHO-DAS 33.3. Hb 7.6 g/dL, MCV 57.9. Brain CT - Mature right striatocapsular infarct with volume loss and minor ex-vacuo distension of the frontal horn of the right lateral ventricle. Retinal imaging - Bilateral WWP		
12 (M/40)	Fever, cough, arthralgia, intense fatigue, **headache, altered consciousness**, vomiting, conjunctivitis (14 d) C_t - value 22.8	(421 d) Eye pain, redness and photophobia. Sharp/shooting pains in limbs with altered sensations. Difficulty sleeping. On examination, conjunctivitis, no joint effusions. Retinal imaging – bilateral extensive peripapillary pale retinal lesions with pigmentation of larger lesions with sparing of the fovea	Uveitis. Large joint, asymmetrical polyarthritis.	Analgesia. Refer to ophthalmology clinic
13 (F/58)	Fever, cough, runny nose, ear ache, chest pain, arthralgia/myalgia, **headache**, shortness of breath, **altered consciousness**, abdominal pain, nausea, vomiting, diarrhea, conjunctivitis (31 d)	(497 d) All over headache associated with blurred/altered vision and eye pains. Reflux symptoms. Generalized weakness and subjective changes in thinking	Undifferentiated headache	Referred back to general survivor's clinic
14 (M/38)	Fever, hemoptysis, sore throat, chest pain, arthralgia/myalgia, headache, shortness of breath, seizures, abdominal pain, nausea, diarrhea, unconsciousness (18 d). C_t value – 24.1	(441 d) Intermittent all over headaches associated with fever, eye pains and photophobia	Possible anterior uveitis Undifferentiated headache	Referral to clinic
15 (F/49)	Fever, cough with sputum, chest pain, arthralgia/myalgia, intense fatigue, **altered consciousness**, abdominal pain, nausea, vomiting, **unconsciousness** (11 d)	(452 d) Headache 2–3 times weekly, constant, band-like. Associated problems with distant vision in left eye. Improves with rest and analgesia	Tension-type headache	Referred back to general survivor's clinic
16 (F/31)	Fever, sore throat, arthralgia/myalgia, **headache**, **altered consciousness**, abdominal pain, nausea, vomiting, diarrhea, **unconscious** −2 weeks (19 d). C_t value – 23.5	(435 d) All over headache, aching, lasting 3–5 h, worse on carrying loads and associated with photophobia and dizziness. MMSE 26/27. WHO-DAS 12.50. Brain CT - Cerebral and cerebellar volume loss. Retinal imaging - Normal fundus	Migraine headache	Propranolol 20 mg daily with improved symptoms (unable to quantify)
17 (F/51)	Fever, hemoptysis, sore throat, runny nose, ear ache, chest pain, arthralgia/myalgia,	(402 d) Headache, no associated symptoms. Altered sensations in feet. Difficulties with doing up buttons. On	Undifferentiated headache Peripheral sensory neuropathy	Referred back to general survivor's clinic

Pt no (Sex/Age)	Acute disease features (Length of stay/days) (Zaire Ebola virus PCR result/Ct value)	Neurologic features at review in clinic - time since acute disease/days, symptoms and examination findings, MMSE and WHO-DAS 2.0 score, selected serum, cerebrospinal fluid radiographic, ECG and retinal investigations	Neurologic/psychiatric diagnosis	Management and outcome
	**headache**, shortness of breath, abdominal pain, nausea, vomiting, diarrhea, conjunctivitis (32 d). C_t value – 22.5	examination, bilateral lower limb altered sensation in stocking distribution in lower limbs		
18 (F/32)	Fever, hemoptysis, ear ache, chest pain, arthralgia/myalgia, **headache**, shortness of breath, **altered consciousness**, **seizures**, abdominal pain, nausea, vomiting, skin rash, **unconsciousness** (37 d)	(448 d) Tinnitus, with improvement of symptoms in specialist clinic. Eye pains, right worse than left. Worse in bright sunlight. Vision normal. Knee pains and chest pains. On examination, conjunctival tenderness. MMSE 21/22. WHO-DAS 18.75. Brain CT - Normal study. Retinal imaging - Right WWP. Left normal fundus	Arthralgia, anterior uveitis	Ophthalmology referral. Analgesia. MH follow up Review at 1 y – improvement in tinnitus, now occasional
19 (M/38)	Fever, heamoptysis, sore throat, chest pain, arthralgia/myalgia, **headache**, abdominal pain, nausea, diarrhea, conjunctivitis, **unconsciousness** (30 d). C_t value – 28.2	(448 d) All over headache triggered when thinks of family passing away. Feels like a pressure in head and vertigo. Reported visual hallucinations, irritability and anxiety. Joint pains. On examination, fixed boutonnieres deformity 3rd and 4th MCP joints. Short of breath on exertion. MMSE 24/26. WHO-DAS 2.08. CXR/ECG normal. Retinal imaging - Normal bilaterally	Undifferentiated headache, arthralgia	Local MH follow-up
20 (F/30)	Fever, hemoptysis, sore throat, runny nose, ear ache, chest pain, arthralgia/myalgia, **headache**, shortness of breath, abdominal pain, nausea, vomiting, diarrhea, conjunctivitis, bleeding (miscarriage) (28 d). C_t value – 30.4	(395 d) Initial presentation with right sided headache with photophobia and scotoma, resolved on review in specialist clinic. MMSE 20/22. WHO-DAS 4.17. RBG 5.0 mmol/L. Brain CT – Normal study. Retinal imaging - Normal bilaterally	Resolved migraine headache, arthralgia	Analgesia. Review at 1 y – new onset headache with cluster-type features
21 (F/32)	Fever, sore throat, runny nose, chest pain, arthralgia/myalgia, intense fatigue, **headache**, shortness of breath, **altered consciousness**, abdominal pain, nausea, vomiting, diarrhea, conjunctivitis, skin rash, **unconscious** - 2 weeks (26 d). C_t value – 21.4	(332 d) Pounding frontal headache. Associated photophobia, phonophobia and scotoma. Occurring approximately monthly. Tinnitus. Eye pain right >left. Now cloudy loss of vision in right eye. On examination right sided corneal opacity. MMSE 26/26. WHO-DAS 8.33. Brain CT – Normal study. Retinal imaging - Right one inferior retinal pigmented lesion. Left normal fundus	Migraine headache, right eye cataract, cataract, arthralgia	Analgesia. Ophthalmology referral
22 (F/21)	Fever, hemoptysis, arthralgia/myalgia, **headache**, shortness of breath, nausea, vomiting, **unconscious** (27 d)	(376 d) Severe, pounding headaches, vertex to occiput with intermittent blurring of vision and dizziness. Photophobia and phonophonia. Occur monthly, lasting 5–7 d. Tinnitus. Subjectively mentally slow for 1 mo post discharge	Migraine headache	Propranolol 20 mg daily. Headache improved 8/10 to 4/10. Review at 1 y – no further migraine symptoms

Pt no (Sex/Age)	Acute disease features (Length of stay/days) (Zaire Ebola virus PCR result/Ct value)	Neurologic features at review in clinic - time since acute disease/days, symptoms and examination findings, MMSE and WHO-DAS 2.0 score, selected serum, cerebrospinal fluid radiographic, ECG and retinal investigations	Neurologic/psychiatric diagnosis	Management and outcome
		and hands trembled doing anything. MMSE 24/30. WHO-DAS 2.08. ESR 22 mm/hr, RBG 4 mmol/L. Brain CT - Normal fundus. Retinal imaging - Normal fundus		
23 (M/46)	Fever, cough with sputum, sore throat, runny nose, ear ache, chest pain, arthralgia/myalgia, intense fatigue, **headache**, shortness of breath, **altered consciousness**, abdominal pain, nausea, vomiting, conjunctivitis, rash (24 d). C_t value – 34.7	(424 d) All over headache associated with dizziness. Bilateral lower limb tremor. On examination, bilaterally lower limb tremor worse on movement. Brain CT - Focus calcification at right globus pallidus. Retinal imaging - Left 3 chorioretinal pigmented lesions. Right chorioretinal lesion emanating from the optic disc, and peripheral pigmented lesion with pigmentation of the retinal vasculature	Essential tremor, undifferentiated headache	DNA specialist clinic
24 (F/43)	Fever, cough, runny nose, chest pain, arthralgia/myalgia, **headache**, shortness of breath, **altered consciousness**, nausea, vomiting, rash (9 d). C_t value – 20.7	(404 d) Headaches since discharge. 2–3x weekly lasting few hours up to 2 d. Frontal, not pounding more like an ache, photophobia and phonophobia. Occasional vomiting, helped by paracetamol. Previous mild headaches. MMSE 30/30. Brain CT – Normal study. Retinal imaging - Intermediate uveitis left eye. Right normal fundus	Migraine headache	Propranolol 20 mg daily, initially 10/10 headache pain now better (not able to quantify). Review at 1 y – decreased frequency of headaches, now occasional
25 (M/42)	Fever, runny nose, ear ache, chest pains, arthralgia/myalgia, intense fatigue, **headache**, shortness of breath, **altered consciousness**, abdominal pain, nausea, vomiting, diarrhea, conjunctivitis (8 d)	(545 d) Sudden onset weakness of left side occurring 4 d post discharge. Speech and comprehension difficulties. Pervasive low mood, anhedonia, feelings of worthlessness, guilt, frustration and hopelessness. Left hemiplegia, hemiasthenia, left homonymous hemianopia. MMSE 26/27. WHO-DAS 89.58. Brain CT - Mature right MCA infarct. Retinal imaging - Bilateral Ebola retinal lesion	Extensive right MCA infarct, major depressive disorder	Physiotherapy, MH follow up. Review at 1 y – improvement in symptoms. Patient subsequently died
26 (F/25)	Fever, heamoptysis, sore throat, runny nose, ear ache, chest pain, arthralgia/myalgia, intense fatigue, **headache**, shortness of breath, abdominal pain, nausea, vomiting, conjunctivitis, rash, **unconscious** (31 d)	(272 d) Pain and weakness right upper limb. Right upper limb; atrophy and 4/5 power in ulnar nerve distribution, no sensory impairment. Brain CT - Small focal calcification left mesial temporal lobe	Ulnar nerve palsy	DNA specialist clinic
27 (M/25)	Fever, cough with sputum, sore throat, runny nose, arthralgia/myalgia, intense fatigue, **headache**, **altered consciousness**, abdominal pain, nausea,	(422 d) Right sided headache with photophobia and phonophobia. Left thigh wasting and 4/5 power left HF/KF. MMSE 29/29. WHO-DAS 6.25. RBG 5.0 mm/L,	Migraine headache, arthralgia	Analgesia, MH follow up. Review at 1 y – decreased frequency of headaches, now occasional

Pt no (Sex/Age)	Acute disease features (Length of stay/days) (Zaire Ebola virus PCR result/Ct value)	Neurologic features at review in clinic - time since acute disease/days, symptoms and examination findings, MMSE and WHO-DAS 2.0 score, selected serum, cerebrospinal fluid radiographic, ECG and retinal investigations	Neurologic/psychiatric diagnosis	Management and outcome
	vomiting, diarrhea, skin rash (11 d). C_t value –29.4	Cholesterol 3.5 mmol/L. Brain CT – Normal study. Retinal imaging - Normal bilaterally		
28 (F/21)	Fever, runny nose, chest pain, arthralgia/myalgia, intense fatigue, **headache**, **altered consciousness**, abdominal pain, nausea, vomiting, diarrhea, skin rash, skin ulcer, **unconscious** - 20 d (14 d)	(455 d) Approximately monthly, frontal, pounding headache, lasting a few hours. Improving symptoms. Currently pregnant 24/42. MMSE 19/26. WHO-DAS 0. Retinal imaging - Bilateral Ebola retinal lesion. Left WWP. Right normal fundus, small posterior subcapsular cataract	Tension-type headache	Analgesia. Review at 1 y - decreased frequency of headaches, now occasional. Lost pregnancy with fever/rash
29 (F/61)	Fever, cough with sputum, sore throat, runny nose, chest pain, intense fatigue, **headache, altered consciousness**, conjunctivitis, **unconsciousness** (12 d). C_t value – 21.0	(403 d) Constant headache, bank-like with associated eye pain and photophobia. Generalized joint pains with longstanding right knee pain. Generalized weakness. Difficulty sleeping and depression. MMSE 28/30. WHO-DAS 12.5. Retinal imaging - Bilateral subcapsular cataract with several pigmented peripheral lesions	Migraine headache, bilateral cataract, arthralgia	Local MH follow up
30 (F/19)	Fever, cough, sore throat, chest pain, arthralgia/myalgia, intense fatigue, **headache**, **altered consciousness**, abdominal pain, nausea, vomiting, rash. 21 d	(502 d) Bilateral red eyes and eye pain with associated constant headache and intermittent fevers. Bilateral knee pains.	Anterior uveitis, undifferentiated headache	Urgent referral to local ophthalmology clinic
31 (F/33)	Fever, ear ache, arthralgia/myalgia, intense fatigue, **headache**, shortness of breath, **altered consciousness**, conjunctivitis, **unconscious** - 2 d (11 d)	(698 d) Headaches for past 5 y, worse since Ebola virus disease. Pounding in forehead. Daily - mainly at night. Mild photophobia and phonophobia. Occasional vomiting. Ongoing feelings of anxiety, heightened when thinking of future and difficulties with work and family, not being able to support them. MMSE 25/30. WHO-DAS 10.42. Brain CT – Normal study. Retinal imaging - Normal bilaterally	Migraine headache, generalized anxiety disorder	Propranolol 20 mg daily, improved headache from 10/10 to 6/10. MH follow up,
32 (F/43)	Fever, cough, sore throat, runny nose, wheeze, chest pain, arthralgia/myalgia, **headache**, shortness of breath, **altered consciousness**, abdominal pain, nausea, vomiting, diarrhea, conjunctivitis, rash (28 d)	(471 d) Cloudy vision, intermittent headache with no added symptoms. Symmetric pains in joints with stiff fingers. On examination, fixed boutonnieres deformity 3rd and 4th MCP joints. Bilateral cataracts	Undifferentiated headache, arthralgia	Referred to local ophthalmology clinic
33 (F/41)	Fever, sore throat, chest pain, arthralgia/myalgia, **headache**, shortness of breath, **altered consciousness**, nausea,	(497 d) Right sided headache, sharp but pounding with noises. Associated photophobia and blurred vision. MMSE 15/21. WHO-DAS 2.08. Brain CT – Normal study.	Migraine headache, arthralgia, anxiety	MH follow up, simple analgesia. Review at 1 y - decreased frequency of

Pt no (Sex/Age)	Acute disease features (Length of stay/days) (Zaire Ebola virus PCR result/Ct value)	Neurologic features at review in clinic - time since acute disease/days, symptoms and examination findings, MMSE and WHO-DAS 2.0 score, selected serum, cerebrospinal fluid radiographic, ECG and retinal investigations	Neurologic/psychiatric diagnosis	Management and outcome
	vomiting, diarrhea, conjunctivitis, rash (25 d)	Retinal imaging - Bilateral WWP		headaches, now occasional
34 (F/25)	Fever, sore throat, chest pain, arthralgia/myalgia, intense fatigue, **headache**, vomiting, nausea, diarrhea (18 d)	(398 d) All over headache with no associated symptoms. Reduced visual acuity right eye	Undifferentiated headache	Referred to general survivor's clinic
35 (M/35)	Fever, sore throat, runny nose, wheeze, chest pain, arthralgia/myalgia, intense fatigue, **headache**, **altered consciousness**, **seizures**, nausea, vomiting, diarrhea, conjunctivitis, rash (28 d)	(515 d) Left sided pounding headache 4 mo after discharge, associated photophobia/phonophobia, occurring approximately monthly. Burning in feet, started 1 y after discharge. Pervasive low mood, difficulty sleeping. On examination, asymmetric glove and stocking peripheral neuropathy, light touch and pinprick >proprioception. MMSE 26/29. WHO-DAS 18.75. Hb 12.6 g/dL, MCV 78.6, ESR 68, Rh F negative. Knee XRs normal. OGTT 8.4 mmol/L - 0 h., 10.1–2 h. Brain CT: Normal study. Retinal imaging, bilateral. Ebola retinal lesions and WWP	Migraine headache, asymmetric sensory peripheral neuropathy, major depressive disorder	MH follow up, Propranolol 20 mg daily, Gabapentin 300 mg nocte, diet and diabetic clinic referral. Headache improved (unable to quantify), pain in feet improved. Review at 1 y: Decreased frequency of headaches, now occasional. Improvement in neuropathy
37 (F/12)	Fever, arthralgia/myalgia, intense fatigue, **altered consciousness**, **seizures**, abdominal pain, diarrhea, conjunctivitis, **unconscious** - 1 mo (15 d). C_t value – 27.9	(454 d) Long period of coma post Ebola virus disease infection. Now deaf and blind with severe cognitive deficit requiring 24-h care. No focal weakness. Cerebrospinal fluid EBoV PCR negative. Brain CT – Significant marked parietal and temporal lobe atrophy	Severe neuro-cognitive impairment post viral encephalitis	Referral to orphanage for 24-h care
38 (M/21)	Fever, cough, sore throat, runny nose, chest pain, arthralgia/myalgia, intense fatigue, **headache**, shortness of breath, **altered consciousness**, abdominal pain, nausea, vomiting (21 d)	(503 d) All over headache with no associated symptoms. Visual hallucinations. Arthralgia	Undifferentiated headache, arthralgia	No data

*Boldface type indicates neurologic features.; MCA, middle cerebral artery; MH, mental health; WWP, white without pressure.

Technical Appendix Table 4. Table showing demographic and clinical features of patients diagnosed with headache attending preliminary and specialist neurology and psychiatric clinics, n = 30*

**Pt no.**	Frequency and duration	Description/ location	Exacerbating and associated factors	History of headache	Other	Diagnosis
1 (M/21)		Throbbing/ pounding. Frontal	Visual disturbance	No	ND	Common migraine
2 (M/47)	Every 2 weeks Lasting up to 1 week	Intense. All over	Scotoma	No	Resolved by time of specialist review	Common migraine (resolved)
4 (M/33)	2 weekly Lasting 1 d – 1 week	Band like	Scotoma, Confusion	No	ND	Common migraine
5 (F/54)	ND	All over	Scotoma Mouth burning	No	ND	Undifferentiated headache
6 (F/18)	ND	All over	-	No	ND	Undifferentiated headache
7 (F/21)	Daily	Constant. Band-like	Difficulty sleeping	No	ND	Tension headache
8 (F/29)	ND	All over	None	No	ND	Undifferentiated headache
9 (F/26)	Daily (at night) Lasting a few hours	Frontal	Blurred vision	No	ND	Common migraine
11 (M/42)	ND	All over	ND	No	Large left MCA stroke	Undifferentiated headache
13 (F/58)	ND	All over	Eye pain Altered vision	No	Subjective changes in thinking	Undifferentiated headache
14 (M/38)	Intermittent	All over	Photophobia Eye pain Fever	No	Possible anterior uveitis	Undifferentiated headache
15 (F/49)	2–3 times weekly	Band-like and constant	ND	No	Problems with vision in left eye	Tension headache
16 (F/31)	Lasting a few hours	Aching. All over	Photophobia Carrying loads Dizziness	No	Onset 4/12 post d/c	Common Migraine
17 (F/51)	ND	All over		No	ND	Undifferentiated headache
19 (M/38)	ND	Pressure. All over	Thinking about loss of family Vertigo	ND	ND	Undifferentiated
20 (F/32)	ND	Right sided	Photophobia Scotoma	No	Resolved by time of specialist review	Common migraine (resolved)
21 (F/32)	Monthly Lasting a few hours	Pounding. Frontal	Photophonia Photophobia Scotoma	No	Poor vision, Amenorrhea	Common Migraine
22 (F/21)	Monthly Lasting 5–7 d	Pounding. Vertex to occiput	Photophonia Photophobia Blurred vision, Dizziness	Maternal history	Started 5 weeks post EVD	Common Migraine
23 (M/46)	ND	All over	Dizziness	No	ND	Undifferentiated headache
24 (F/43)	2–3x weekly Lasting a few hours up to 2 d	Aching. Frontal	Photophonia Photophobia Occ. vomiting	Yes. Previously mild, worse since EVD	Intermedate uveitis left eye	Common migraine
27 (M/25)	2–3x weekly Lasting a few hours	Pounding. Right sided	Photophonia Photophobia Blurred vision	No	ND	Common Migraine
28 (F/21)	Monthly Lasting a few hours	Pounding. Frontal	ND	No	Pregnant 24/42 weeks. Improving symptoms	Tension headache
29 (F/19)	Constant	Bank-like	Photophobia Eye pain Difficulty sleeping	No	ND	Undifferentiated headache

**Pt no.**	Frequency and duration	Description/ location	Exacerbating and associated factors	History of headache	Other	Diagnosis
30 (F/33)	Constant		Bilateral red eyes with pain Intermittent fevers	ND	Anterior uveitis	Undifferentiated headache
31 (F/33)	Daily worse at night Up to 4 h	Pounding. Frontal	Photophonia Photophobia Occ. Vomiting	5 y, worse since EVD	ND	Common Migraine
32 (F/43)	Intermittent	All over	ND	No	Cloudy vision	Undifferentiated headache
33 (F/41)	Daily	Sharp becomes pounding. R sided	Photophonia, Photophobia Blurred vision and vertigo	No	ND	Common Migraine
34 (F/25)	ND	All over	Reduced acuity right eye	ND	ND	Undifferentiated headache
35 (M/35)	Monthly	Pounding. Left sided	Photophonia Photophobia Difficulty sleeping	No	ND	Common Migraine
38 (M/21)	ND	All over	ND	No	ND	Undifferentiated

*ND, no data.

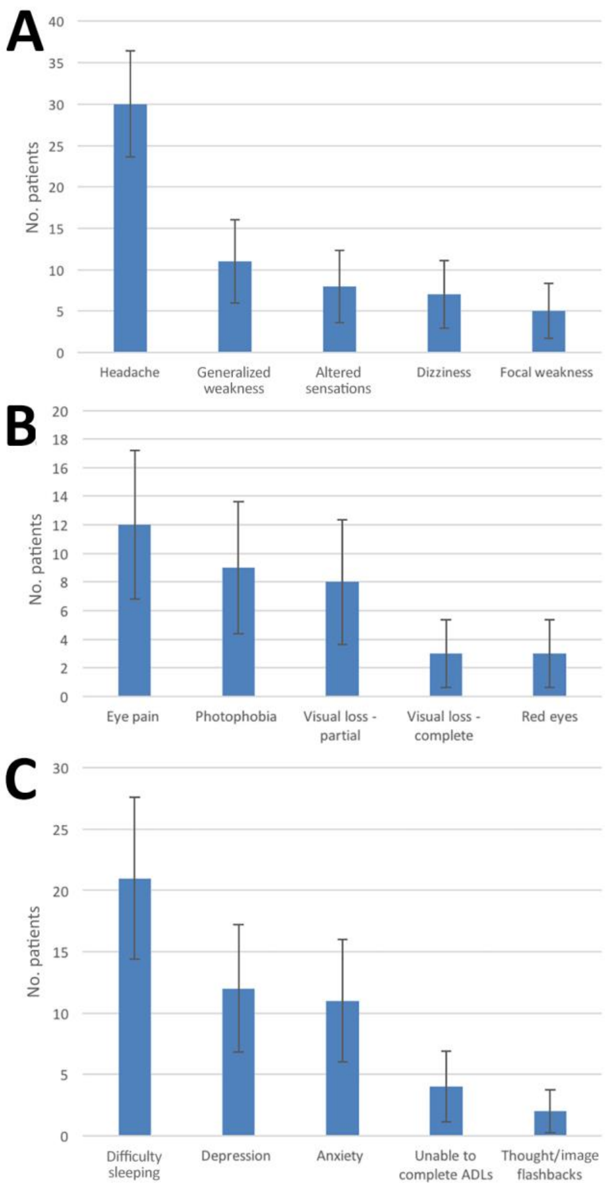

Technical Appendix Figure. Figure showing series of 3 histograms showing frequency of symptoms among patients attending the preliminary clinic (n=40) who had specific A) neurologic, B) ophthalmologic, and C) psychiatric symptoms. Error bars indicate 95% CI. ADL, activities of daily living.
